# AGE AND OCCUPATIONAL TIME PERSPECTIVE ARE ASSOCIATED WITH PREFERENCE FOR HELPING OTHERS AT WORK

**DOI:** 10.1093/geroni/igz038.465

**Published:** 2019-11-08

**Authors:** Yochai Z Shavit, Laura L Carstensen

**Affiliations:** Stanford University, Stanford, California, United States

## Abstract

Workforces today are more age-diverse than ever before. Despite widespread beliefs that older workers are less productive than younger workers, when productivity is measured at the team level, the presence of older workers appears to positively contribute to productivity. Underlying reasons for this finding have been poorly understood. Socioemotional selectivity theory (SST) posits that goals change with age and that when time-horizons grow increasingly limited people prefer activities that hold meaning in their pursuit, such as helping others. Reasoning from SST, we hypothesized that age is associated with a preference for helping colleagues. We further hypothesized that expanding time-horizons would reduce age differences such that both younger and older people would prefer projects that help colleagues. 555 workers, aged 20-75, were assigned to one of three experimental conditions where time-horizons were expanded, constrained or not mentioned (control), and were asked to indicate their preferences for projects that benefitted themselves or others. Results from multinomial logistic regression show that collapsing across conditions, age was positively associated with a preference for projects helping friends or colleagues over ones that were career advancing, and participants were more likely to work on a helping project over one that would take the company in a new direction regardless of age. We further found effects of manipulating time horizons- in both the expanded and limited occupational time-horizons conditions workers were less likely to prefer helping compared to advancing their careers, and the positive association between age and preference for helping was attenuated.

